# Sleep profiles of different psychiatric traits

**DOI:** 10.1038/s41398-024-03009-4

**Published:** 2024-07-12

**Authors:** John Axelsson, Eus J. W. van Someren, Leonie J. T. Balter

**Affiliations:** 1 Department of Clinical Neuroscience, Karolinska Institutet, Stockholm, Sweden; 2 Department of Psychology, Stress Research Institute, Stockholm University, Stockholm, Sweden; 3 Department of Sleep and Cognition, Netherlands Institute for Neuroscience, Amsterdam, The Netherlands; 4 Department of Integrative Neurophysiology and Psychiatry, Center for Neurogenomics and Cognitive Research, Amsterdam UMC, Amsterdam Neuroscience, VU University, Amsterdam, The Netherlands

**Keywords:** Predictive markers, Human behaviour

## Abstract

Disturbed sleep comes in many forms. While the key role of sleep in mental health is undisputed, our understanding of the type of sleeping problems that manifest in the early stages of psychiatric disorders is limited. A sample without psychiatric diagnoses (*N* = 440, 341 women, 97 men, 2 non-binaries; *M*_age_ = 32.1, *SD* = 9.4, range 18–77) underwent a comprehensive assessment, evaluating eight sleep features and 13 questionnaires on common psychiatric complaints. Results revealed that traits of affect disorders, generalized anxiety, and ADHD had the worst sleep profiles, while autism disorder, eating disorder, and impulsivity traits showed milder sleep issues. Mania was the only trait associated with an overall better sleep profile. Across traits, insomnia and fatigue dominated and sleep variability was least prominent. These findings provide support for both transdiagnostic and disorder-specific targets for prevention and treatment.

## Introduction

Sleep disturbances are common among individuals with subclinical and clinical psychiatric disorders, contributing to the development or worsening of symptoms [1, 2]. Sleep disturbances can manifest in various forms, including insomnia, hypersomnia, and alterations in sleep duration and rhythms [2, 3]. Insomnia or hypersomnia is explicitly listed as diagnostic criterion in disorders such as major depressive disorder (MDD), generalized anxiety disorder (GAD), and posttraumatic stress disorder (PTSD), while a decreased sleep need is typical in the manic phase of bipolar disorder [4]. Sleep disturbances are also prevailing across a spectrum of other disorders from attention-deficit hyperactivity disorder (ADHD) [5] to schizophrenia [6]. Moreover, they are frequently present in at-risk populations and subthreshold phenotypes, and correlate with psychiatric symptoms in otherwise healthy individuals [7–9]. Other sleep-related features that have been associated with mental problems include being an evening chronotype or having a delayed sleep phase (i.e., late sleep and wake times), and social jetlag (i.e., misalignment between an individual’s circadian rhythm and their external social or work schedules) [10–12]. Given the potential for various effective treatments [2, 13, 14], identifying the most prominent sleep features in different mental health presentations represents a fruitful research target.

Important steps in guiding prevention strategies include a better understanding of the type of sleep features that characterize populations with psychiatric symptoms but lacking formal diagnoses. Constructing sleep feature profiles can indicate which sleep features may contribute to symptomatology and can identify potential risk factors and prevention targets before disorder onset. Analyzing the same individuals across all sleep and psychiatric dimensions enables us to gauge the magnitude of associations relative to each other.

Dimensional approaches involve studying a spectrum of underlying causes and mental health issues rather than focusing on clinical populations [15]. Instead of focusing on mental health conditions as discrete categories, dimensional approaches recognize that mental health exists on a continuum or spectrum, with varying degrees of severity. This approach can offer valuable insights into the mechanisms involved in the early stage of psychiatric disorders and facilitate a better understanding of the complex interplay between sleep and psychiatric problems, potentially leading to more effective preventative strategies [16].

Using a dimensional approach, we aimed to identify the most central sleep features seen in different psychiatric dimensions in a cohort free of formal psychiatric diagnoses. Based on literature on the presence of sleep symptoms in disorders and literature on the predictive value of sleep and circadian features in longitudinal risk studies, we hypothesized that insomnia, fatigue, and the evening chronotype would be the most prominent, particularly in traits relating to mood disorders. A better understanding of the specific sleep features associated with mental health can aid prevention and tailoring treatment strategies to address central sleep issues associated with psychiatric problems.

## Methods

### Participants

A total of 440 participants (341 women, 97 men, 2 non-binary; *M*_age_ = 32.1, *SD* = 9.4, range 18–77) were included in the final analyses after excluding 75 individuals with a psychiatric diagnosis, taking psychotropic medication, or incorrectly answering the data quality checks. Among the 440 participants, 273 (62.0%) worked part-time, full-time, or were self-employed, 77 (17.5%) were part-time or full-time students, 6 (1.4%) did voluntary work, 47 (10.7%) were unemployed, 2 (0.5%) were retired, and 18 (4.1%) did not fall into any of these categories (e.g., homemaker/parent, in between jobs, sick leave) (the total can add up to > 100% due to an individual fitting multiple categories).

### Recruitment strategy

Participants were recruited via an online recruitment platform (Prolific.co). Individuals can sign up for studies that are listed on this platform. Researchers can specify the eligibility criteria for their studies. Participants qualified if: residing in United Kingdom; fluent in English; ≥18 years; ≥99% approval of previous participations on Prolific.co. Individuals with a psychiatric diagnosis or taking psychotropic medication were excluded (see also under “Participants”). No other stringent exclusion criteria applied in order to recruit participants with a diverse range of psychiatric trait levels, ranging from low level to high level symptoms. Participants received financial compensation. See Supplement for further information on recruitment.

### Procedures and measures

The study is part of a larger two-day study on diurnal variation in psychiatric symptoms (Balter et al.^17^ it also includes the chronotype-psychiatric trait associations but including a larger sample than the one used here) and cognitive functioning. The data analyzed in the present study were collected during the baseline session. The baseline session was conducted on a weekday between 09:00-21:00. Participants completed questionnaires on sleep, psychiatric traits and risk factors, and performed brief cognitive tests (results not reported here). Data collection took place in October 2021, during the COVID-19 pandemic.

#### Ethics approval

All participants provided online informed consent at the start of the study. The study was approved by the Swedish Ethical Review Authority (dnr:2021-01695) and performed in accordance with ethical principles of the Declaration of Helsinki.

#### Sleep features

The following sleep features were assessed: sleep duration deviation (the participant’s deviation from the sample’s mean sleep duration was calculated for the last night’s sleep, represented as either shorter or longer than the mean of 7h39min); fatigue (one item of the Sickness Questionnaire [18]); social jetlag (Munich Chronotype Questionnaire [19]); non-restorative sleep; poor sleep quality; perceiving sleep as insufficient (Karolinska Sleep Questionnaire [20]); evening chronotype (reduced Morningness Eveningness Questionnaire [21]); insomnia (Insomnia Severity Index [22]). The KSQ and the ISI were not administered in a subset of the participants. See Supplement for further information. These features were selected as they represent central dimensions of sleep and encompass a spectrum of sleep features commonly encountered to deviate in psychiatric disorders [2, 23, 24]. The inclusion of chronotype and social jetlag was deemed relevant due to their potential impact on circadian rhythms and its association with certain psychiatric symptoms [10, 23, 25].

#### Psychiatric trait questionnaires

Thirteen validated questionnaires on common psychiatric traits and risk factors were included (referred to as “psychiatric traits” for the remainder of the text) assessing: depression; generalized anxiety; mania; delusional ideation; emotion dysregulation; autism; impulsivity; emotional instability; ADHD; obsessive compulsive disorder (OCD); eating disorder; apathy; social anxiety. See Table 1 for an overview of all questionnaires and the sample sizes per measure. The questionnaires were completed in the order as listed in Table 1 unless otherwise stated. Demographic information, as well as details pertaining to psychiatric diagnoses and medication intake were also collected. See Supplement for further information about the sample, including sample distribution plots and heatmaps of the questionnaires.

**Table 1:** Summary of the sleep features and psychiatric trait measures, along with their respective sample sizes (N).

Sleep feature measures	Main outcome	N
Time turned off light, minutes to fall asleep, time woken up	Last night’s sleep duration, sleep duration deviation (shorter or longer sleep than the sample mean)	439/436
Munich Chronotype Questionnaire (MCTQ) [19]	Social jetlag	436
Karolinska Sleep Questionnaire (KSQ) complaints [20]	Non-restorative sleep, poor sleep quality, perceived too little sleep	377
Reduced Morningness-Eveningness Questionnaire (rMEQ) [21]	Chronotype	440
Insomnia Severity Index (ISI) [22]	Insomnia	375
“I feel tired” item of the Sickness Questionnaire^a^ [18]	Fatigue	440

Psychiatric trait questionnaires	Main outcome	N
Center for Epidemiologic Studies Depression Scale Revised Short Form (CESD-R 10) [34]	Depression trait	440
Generalized Anxiety Disorder-7 (GAD-7) [35]	Generalized anxiety trait	440
Altman Self-Rating Mania Scale (ASRMS) [36]	Mania trait	440
Peters Delusions Inventory 21 (yes/no subscale) (PDI-21) [37] and unusual experiences subscale of the Oxford-Liverpool Inventory of Feelings and Experiences (O-LIFE) [38]	Delusional ideation trait	440
Difficulties in Emotion Regulation Scale-16 (DERS-16) [39]	Emotion dysregulation trait	440
Autism Quotient-10 (AQ-10) [40]	Autism trait	440
Health-relevant Personality Inventory (HP5i) impulsivity subscale [41]	Impulsivity trait	440
Affective Lability Scale (ALS-18) [42]	Emotional instability trait	440
Adult ADHD Self-Report Scale (ASRS) [43]	ADHD trait	440
Obsessive Compulsive Inventory-Revised (OCI-R) [44]	OCD trait	440
Eating Attitudes Test-26 (EAT-26) part B [45]	Eating disorder trait	440
Apathy Evaluation Scale (AES) [46]	Apathy trait	440
Liebowitz Social Anxiety Scale (LSAS) [47]	Social anxiety trait	440

*Note*. Questionnaires were completed in the order as presented. *ADHD* Attention Deficit Hyperactivity Disorder, *OCD* Obsessive Compulsive Disorder. See Figure S3 for distribution plots of the psychiatric trait questionnaires.

^a^The fatigue measure was completed after the PDI-21 and O-LIFE subscale.

#### Data quality checks

For data quality purposes, two attention checks and one honesty check were included [26]: “Please rate the response alternative ‘agree (9)’ for this question”; “Please answer 100”; “Have you been completely honest in your answers?”. Data of participants who failed more than one quality check (*n* = 6) were excluded.

### Statistical analysis

Univariate fixed effect regression models were fitted to assess the relationship between sleep features and psychiatric traits, using the *lm* function in R. All variables were Z-transformed before analysis to allow comparison of coefficients. For all sleep measures, datapoints > 4 SD above or below the means were removed. This resulted in excluding eight out of 3,705 datapoints (0.22%): four datapoints for social jetlag; one for last night’s sleep duration; an additional three for sleep duration deviation. No datapoints were excluded for other sleep measures.

## Results

### Insomnia

**Fig. 1:**
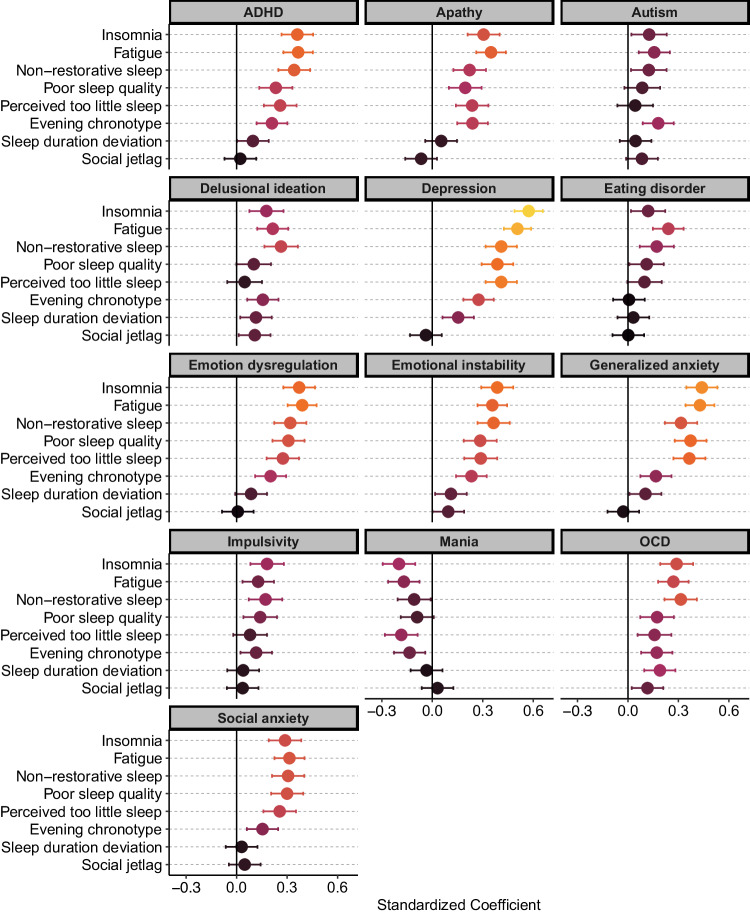
Sleep problem profiles in 13 psychiatric traits. Standardized coefficient plots illustrating the relationships between sleep features and psychiatric traits. Error bars represent 95% confidence intervals. A coefficient of 0 indicates no relationship (range −1 to +1). Lighter color intensities indicate a stronger coefficient. Higher values of sleep duration deviation indicate either a shorter or longer sleep duration than the mean of 7h39min. The rMEQ score was rescored such that a higher score is interpretated as a tendency towards being an evening-type.

Insomnia was among the most dominant sleep problem, associated positively with all traits except for mania, which showed a negative association. See Fig. 1 and Tables S2–14 for results. Traits relating to affect disorders, such as depression, generalized anxiety, emotional instability, and emotion dysregulation, and ADHD were most strongly associated with insomnia. Autism disorder, eating disorder, impulsivity, and delusional ideation traits showed the weakest association with insomnia.

### Fatigue

All traits were associated with fatigue. Fatigue was particularly prominent among affect disorder-related traits. Impulsivity and autism exhibited a weak association with fatigue, and mania was associated with reduced fatigue.

### Non-restorative sleep

Among the sleep features experienced in delusion ideation and OCD, non-restorative sleep was most prominent.

### Poor sleep quality

Poor sleep quality was most prominent in traits of depression and generalized anxiety, followed by emotion dysregulation, social anxiety, emotional instability, ADHD, and apathy. Poor sleep quality was less pronounced in OCD, impulsivity, and eating disorder, and non-significant in delusional ideation, autism, and mania.

### Perceived too little sleep

Mania was associated with a reduced perception of too little sleep. Except for autism, delusional ideation, and impulsivity, all traits were associated with perceiving sleep as insufficient.

### Sleep duration deviation

OCD, depression, delusional ideation, emotional instability, generalized anxiety, and ADHD were the only traits associated with a deviation in sleep duration (i.e., either shorter or longer sleep duration than the sample mean of 7h39min).

### Evening type

Mania was associated with morningness while all other traits (except for eating disorder) were associated with eveningness. See also [17]. In autism disorder, eveningness was the most prominent sleep feature.

### Social jetlag

Modest relationships were apparent between psychiatric traits and the degree of social jetlag, significantly so for OCD, delusional ideation, and emotional instability.

## Discussion

In the present study we characterized the sleep features manifesting in a range of psychiatric aspects in a sample of individuals without formal psychiatric diagnoses. Insomnia was not only strongly related to levels of depression and generalized anxiety, but also to many other psychiatric traits, ranging from features of affect disorders, including apathy, emotion dysregulation, emotional instability, and social anxiety, to ADHD, delusional ideation, and OCD. This mirrors observations in patients diagnosed with a psychiatric disorder, where insomnia disorder is highly common [27]. Given that these associations exist in a population without formal psychiatric diagnoses, it is crucial to evaluate presence of insomnia symptoms in relation to most psychiatric indications, recognizing it as both a risk factor and potential early intervention target. Notably, insomnia treatment yields more favorable outcomes than depression treatment in patients with comorbid depression and insomnia [28]. Moreover, addressing insomnia has shown promise in ameliorating a range of psychiatric symptoms [29], indicating that insomnia interventions could be fruitful for various psychiatric indications.

Traits relating to affect disorders, generalized anxiety, and ADHD showed the worst sleep profiles. Individuals with higher levels of these traits suffered from insomnia, fatigue, non-restorative sleep, poor sleep quality, and insufficient sleep and have an evening chronotype. Mania was the only trait associated with an overall better sleep profile, e.g., lower levels of insomnia, less fatigue, less non-restorative sleep and perceiving sleep as less insufficient, as well as having a morning chronotype. Indeed, a decreased sleep need is among the diagnostic criteria for the manic phase of bipolar disorder [4], which appears to manifest even at subclinical mania levels, as shown in the current study. Autism, eating disorder, and impulsivity showed the least severe sleep profiles, with insomnia, fatigue, or the evening chronotype being their most prominent feature. Delusional ideation and OCD showed a moderately bad sleep profile, with non-restorative sleep being their main sleep complaint. Across all traits, social jetlag was the least significant problem. Despite the eveningness being common among most psychiatric traits (11 out of 13 psychiatric traits), it did not rank among the three most common characteristics in most psychiatric traits (with exceptions noted for autism and apathy). This further highlights the relevance of evaluating multiple sleep characteristics in order to understand the magnitude of their associations relative to each other.

While insomnia is associated with most traits, delusional ideation, eating disorder, and OCD traits showed stronger associations with fatigue and non-restorative sleep. This indicates that various psychiatric vulnerabilities may be best targeted by different sleep intervention strategies. Early identification and management of sleep problems have the potential to mitigate the development or worsening of mental health issues. Tailored sleep intervention strategies, based on the specific sleep profiles, hold significant promise to guide the choice of interventions an individual may benefit from most, although further investigation is necessary. Furthermore, certain combinations of sleep features may indicate a common underlying disturbance. For instance, individuals with elevated OCD symptoms, that was associated with both social jetlag and evening chronotype, may benefit from interventions addressing circadian misalignment - referring to a discrepancy between the body’s internal clock and environmental cues - such as optimal scheduling of light exposure [30]. Conversely, insomnia and fatigue emerged as primary complaints in depression, suggesting these individuals may derive greater benefits from cognitive behavioral therapy for insomnia (CBT-I) treatment [31].

A limitation of this study is the use of self-report measures, which can introduce response bias and require recognition and communication of symptoms. However, many psychiatric symptoms are first and foremost subjective experiences and diagnosis of psychiatric disorders primarily rely on self-reported symptoms and observations. The use of validated measures in this study ensures a standardized and reliable assessment of traits relating to psychiatric disorders. Another limitation is that the cross-sectional design does not allow for conclusions on causality. Furthermore, data were collected during the COVID-19 pandemic, which may have altered sleep patterns [32, 33]. Future research may therefore aim to replicate the findings in a post-pandemic context. Despite these limitations, the thorough comparison of a large range of sleep features and key psychiatric dimensions offers valuable insights into the relationship between sleep characteristics and psychiatric traits within the non-diagnosed range. Analyzing the same individuals across all sleep and psychiatric dimensions allowed for the estimation and illustration of the magnitude of associations with sleep features relative to each other. This provides important information that may help the reader to interpret other research findings in the field by highlighting the relative importance of different sleep health problems. The findings underscore that in populations free of formal psychiatric diagnoses, discernible sleep problems are already noticeable, with insomnia symptoms and fatigue being the prominent problem. The data also show that, in many cases, more than one sleep health problem exists with some differences in the primary, most taxing, problem. Besides the strong relationships seen between insomnia and features of affect disorders, our study highlights that individuals with subclinical, or undiagnosed, delusional ideation and autism display distinct sleep problem profiles, with stronger associations with fatigue, non-restorative sleep, and having an evening chronotype. The findings hold promise for identifying early indicators and potential risk factors for the onset of psychiatric disorders.

### Supplementary information

Supplemental Material

## Data Availability

A markdown file containing the analysis is available on the Open Science Framework (OSF) at https://osf.io/82d9b.

## References

[1] Hertenstein E, Feige B, Gmeiner T, Kienzler C, Spiegelhalder K, Johann A (2019). Insomnia as a predictor of mental disorders: A systematic review and meta-analysis. Sleep Med Rev.

[2] Freeman D, Sheaves B, Waite F, Harvey AG, Harrison PJ (2020). Sleep disturbance and psychiatric disorders. Lancet Psychiatry.

[3] Baglioni C, Battagliese G, Feige B, Spiegelhalder K, Nissen C, Voderholzer U (2011). Insomnia as a predictor of depression: A meta-analytic evaluation of longitudinal epidemiological studies. J Affect Disord.

[4] American Psychiatric Association. Diagnostic and statistical manual of mental disorders, fifth edition, text revision. American Psychiatric Association: Washington, 2022.

[5] Becker SP (2020). ADHD and sleep: recent advances and future directions. Curr. Opin. Psychol..

[6] Ferrarelli F (2021). Sleep Abnormalities in Schizophrenia: State of the Art and Next Steps. Am J Psychiatry.

[7] Marin L, Guàrdia A, González-Rodríguez A, Haba-Rubio J, Natividad M, Bosch E (2023). Sleep Disturbances in At-Risk Mental States and First Episode of Psychosis: A Narrative Review on Interventions. Clocks Sleep.

[8] Gould CE, Karna R, Jordan J, Kawai M, Hirst R, Hantke N (2018). Subjective but Not Objective Sleep is Associated with Subsyndromal Anxiety and Depression in Community-Dwelling Older Adults. Am J Geriatr Psychiatry.

[9] Lunsford-Avery JR, Gonçalves B da SB, Brietzke E, Bressan RA, Gadelha A, Auerbach RP (2017). Adolescents at clinical-high risk for psychosis: Circadian rhythm disturbances predict worsened prognosis at 1-year follow-up. Schizophr Res.

[10] Taylor BJ, Hasler BP (2018). Chronotype and Mental Health: Recent Advances. Curr Psychiatry Rep.

[11] Norbury R (2021). Diurnal preference and depressive symptomatology: a meta-analysis. Sci Rep.

[12] Coogan AN, McGowan NM (2017). A systematic review of circadian function, chronotype and chronotherapy in attention deficit hyperactivity disorder. ADHD Atten Deficit Hyperact Disord.

[13] Furukawa Y, Sakata M, Yamamoto R, Nakajima S, Kikuchi S, Inoue M (2024). Components and Delivery Formats of Cognitive Behavioral Therapy for Chronic Insomnia in Adults: A Systematic Review and Component Network Meta-Analysis. JAMA Psychiatry.

[14] van Maanen A, Meijer AM, van der Heijden KB, Oort FJ (2016). The effects of light therapy on sleep problems: A systematic review and meta-analysis. Sleep Med Rev.

[15] Widiger TA, Samuel DB (2005). Diagnostic categories or dimensions? A question for the Diagnostic and Statistical Manual of Mental Disorders - Fifth Edition. J Abnorm Psychol.

[16] Buysse DJ (2014). Sleep health: can we define It? does it matter?. Sleep.

[17] Balter LJT, Holding BC, Petrovic P, Axelsson J (2024). The rhythm of mental health: the relationship of chronotype with psychiatric trait dimensions and diurnal variation in psychiatric symptoms. Transl Psychiatry.

[18] Andreasson A, Wicksell RK, Lodin K, Karshikoff B, Axelsson J, Lekander M (2018). A global measure of sickness behaviour: Development of the Sickness Questionnaire. J Health Psychol.

[19] Roenneberg T, Wirz-Justice A, Merrow M (2003). Life between clocks: Daily temporal patterns of human chronotypes. J Biol Rhythms.

[20] Nordin M, Åkerstedt T, Nordin S (2013). Psychometric evaluation and normative data for the karolinska sleep questionnaire. Sleep Biol Rhythms.

[21] Natale V, Esposito MJ, Martoni M, Fabbri M (2006). Validity of the reduced version of the Morningness-Eveningness Questionnaire. Sleep Biol Rhythms.

[22] Bastien CH, Vallières A, Morin CM (2001). Validation of the insomnia severity index as an outcome measure for insomnia research. Sleep Med.

[23] Walker WH, Walton JC, DeVries AC, Nelson RJ (2020). Circadian rhythm disruption and mental health. Transl Psychiatry.

[24] Van Someren EJW (2021). Brain mechanisms of insomnia: New perspectives on causes and consequences. Physiol Rev.

[25] Roenneberg T (2023). How can social jetlag affect health?. Nat Rev Endocrinol.

[26] Oppenheimer DM, Meyvis T, Davidenko N (2009). Instructional manipulation checks: Detecting satisficing to increase statistical power. J Exp Soc Psychol.

[27] Dolsen MR, Asarnow LD, Harvey AG (2014). Insomnia as a Transdiagnostic Process in Psychiatric Disorders. Curr Psychiatry Rep.

[28] Blom K, Jernelöv S, Kraepelien M, Bergdahl MO, Jungmarker K, Ankartjärn L (2015). Internet treatment addressing either insomnia or depression, for patients with both diagnoses: A randomized trial. Sleep.

[29] Freeman D, Sheaves B, Goodwin GM, Yu LM, Nickless A, Harrison PJ (2017). The effects of improving sleep on mental health (OASIS): a randomised controlled trial with mediation analysis. Lancet Psychiatry.

[30] Faulkner SM, Bee PE, Meyer N, Dijk D-J, Drake RJ (2019). Light therapies to improve sleep in intrinsic circadian rhythm sleep disorders and neuro-psychiatric illness: A systematic review and meta-analysis. Sleep Med Rev.

[31] Hertenstein E, Trinca E, Wunderlin M, Schneider CL, Züst MA, Fehér KD (2022). Cognitive behavioral therapy for insomnia in patients with mental disorders and comorbid insomnia: A systematic review and meta-analysis. Sleep Med Rev.

[32] Raman S, Coogan AN (2022). Effects of societal-level COVID-19 mitigation measures on the timing and quality of sleep in Ireland. Sleep Med.

[33] Ceolin C, Limongi F, Siviero P, Trevisan C, Noale M, Catalani F (2024). Changes in sleep duration and sleep Timing in the General Population from before to during the First COVID-19 Lockdown: A Systematic Review and Meta-Analysis. Int J Environ Res Public Health.

[34] Cole JC, Rabin AS, Smith TL, Kaufman AS (2004). Development and validation of a Rasch-derived CES-D short form. Psychol Assess.

[35] Löwe B, Decker O, Müller S, Brähler E, Schellberg D, Herzog W (2008). Validation and standardization of the generalized anxiety disorder screener (GAD-7) in the general population. Med Care.

[36] Altman EG, Hedeker D, Peterson JL, Davis JM (1997). The altman self-rating Mania scale. Biol Psychiatry.

[37] Peters E, Joseph S, Day S, Garety P (2004). Measuring delusional ideation: The 21-item Peters et al. Delusions Inventory (PDI). Schizophr Bull.

[38] Mason O, Claridge G (2006). The Oxford-Liverpool Inventory of Feelings and Experiences (O-LIFE): further description and extended norms. Schizophr Res.

[39] Bjureberg J, Ljótsson B, Tull MT, Hedman E, Sahlin H, Lundh LG (2016). Development and validation of a brief version of the difficulties in emotion regulation scale: the DERS-16. J Psychopathol Behav Assess.

[40] Booth T, Murray AL, McKenzie K, Kuenssberg R, O’Donnell M, Burnett H (2013). Brief report: an evaluation of the AQ-10 as a brief screening instrument for asd in adults. J Autism Dev Disord.

[41] Gustavsson JP, Jönsson EG, Linder J, Weinryb RM (2003). The HP5 inventory: definition and assessment of five health-relevant personality traits from a five-factor model perspective. Personal Individ Differ.

[42] Oliver MNI, Simons JS (2004). The affective lability scales: development of a short-form measure. Personal Individ Differ.

[43] Adler LA, Spencer T, Faraone SV, Kessler RC, Howes MJ, Biederman J (2006). Validity of pilot adult ADHD Self-Report Scale (ASRS) to rate adult ADHD symptoms. Ann Clin Psychiatry.

[44] Foa EB, Huppert JD, Leiberg S, Langner R, Kichic R, Hajcak G (2002). The obsessive-complusive inventory: development and validation of a short version. Psychol Assess.

[45] Garner DM, Bohr Y, Garfinkel PE (1982). The eating attitudes test: psychometric features and clinical correlates. Psychol Med.

[46] Marin RS, Biedrzycki RC, Firinciogullari S (1991). Reliability and validity of the apathy evaluation scale. Psychiatry Res.

[47] Liebowitz MR. Liebowitz Social Anxiety Scale. Mod Probl Pharmapsychiatry. 1987.

