# Supplementary material for: Sleep profiles of different psychiatric traits
Source: Transl Psychiatry. 2024 Jul 12;14:284. doi: 10.1038/s41398-024-03009-4 (PMC11245526; doi:10.1038/s41398-024-03009-4)
Supplement: Supplementary file 1 — Supplemental Material [file 41398_2024_3009_MOESM1_ESM.docx]

**SUPPLEMENTARY MATERIAL**

**Sleep profiles of different psychiatric traits**

**Axelsson, van Someren, & Balter**

|  | **Page** |
| --- | --- |
| Supplementary Methods |  |
| Recruitment | [2](#_Recruitment) |
| Drop-out and exclusion | [2-3](#_Drop-out_and_exclusion) |
| Measures | [3-7](#_Measures) |
|  |  |
| **Additional results** |  |
| Table S1: Sample characteristics: smoking behavior and alcohol intake | [8](#_Table_S1._Sample) |
|  |  |
| **Supplementary Tables** |  |
| Table S2: Regression results for the sleep features and ADHD (ASRS) | [9](#_Table_S2._Regression) |
| Table S3: Regression results for the sleep features and apathy (AES) | [10](#_Table_S3._Regression) |
| Table S4: Regression results for the sleep features and autism (AQ-10) | [10](#_Table_S4._Regression) |
| Table S5: Regression results for the sleep features and delusional ideation (PDI-21 &  O-LIFE subscale) | [11](#_Table_S5._Regression) |
| Table S6: Regression results for the sleep features and depression (CESD-R 10) | [11](#_Table_S6._Regression) |
| Table S7: Regression results for the sleep features and eating disorder (EAT-26 part B) | [12](#_Table_S7._Regression) |
| Table S8: Regression results for the sleep features and emotion dysregulation (DERS-  16) | [12](#_Table_S8._Regression) |
| Table S9: Regression results for the sleep features and emotional instability (ALS-18) | [13](#_Table_S9._Regression) |
| Table S10: Regression results for the sleep features and generalized anxiety (GAD-7) | [13](#_Table_S10._Regression) |
| Table S11: Regression results for the sleep features and impulsivity (HP5i) | [14](#_Table_S11._Regression) |
| Table S12: Regression results for the sleep features and mania (ASRMS) | [14](#_Table_S12._Regression) |
| Table S13: Regression results for the sleep features and OCD (OCI-R) | [15](#_Table_S13._Regression) |
| Table S14: Regression results for the sleep features and social anxiety (LSAS) | [15](#_Table_S14._Regression) |
|  |  |
| **Supplementary Figures** |  |
| Figure S1: Frequency plots of the scores of the sleep features | [16](#_Figure_S1._Frequency) |
| Figure S2: Correlation matrix of the sleep features | [17](#_Figure_S2._Correlation) |
| Figure S3: Frequency plots of the scores of the psychiatric traits | [18](#Figure S3. Frequency plots of the scores of the psychiatric trait levels. The black line denotes the available cut-off scores for potential clinical-level symptoms: Depression ( 10; Generalized anxiety ( 10 (= moderate anxiety); Mania ( 6; Autism ( 6; OCD) |
| Figure S4: Correlation matrix of the psychiatric traits | [19](#_Figure_S4._Correlation) |
|  |  |
| **References** | [20-21](#_REFERENCES) |
|  |  |

# Recruitment

To assess feasibility and dropout rates we initially recruited 65 participants. The sleeping problems item of the KSQ and the ISI were not administered in this subset. The inclusion criterion of having a 99% or higher approval rate in previous participations on Prolific.co refers to the percentage of completed tasks that were approved on the platform. This serves as an indicator of the participant’s consistent adherence to study requirements and expectations as set by the researchers. Recruiting participants through online platforms such as Prolific allow access to a diverse participant pool, potentially reducing logistical, physical, and/or mental constraints for participating in research. A limitation to consider is the limited control over the participant’s surrounding. Variations in factors such as noise levels, distractions, and technological issues may impact data quality and consistency. Prolific, compared to Amazon Mechanical Turk, CloudResearch, Qualtrics, and Dynata, has in evaluations been judged to be the only platform that provides high data quality across all assessed data quality dimensions, including attention, comprehension, honesty, and reliability ^1^.

We choose not to implement stringent exclusion to capture a diverse range of psychiatric trait levels, spanning from low-level to high-level symptoms. Nonetheless, we collected information regarding psychiatric diagnoses and medication intake. Participants who reported one or more current psychiatric diagnoses or using psychotropic medications were excluded after the data collection phase for the objectives of this study.

# Drop-out and exclusion

The baseline session was completed by 515 individuals. For data quality purposes, two attention checks and one honesty check were included in the baseline sessions ^2^. The first attention check was positioned in the first quarter of the study (“Please rate the response alternative 'agree (9)' for this question”), followed by the second attention check midway through (“Please answer 100”), and the honesty question at the end of the study (“Have you been completely honest in your answers?”). The position of these items was fixed for all participants. Baseline data of participants who failed more than one quality check (*n* = 6) were excluded before analysis. Failure was defined as not selecting “agree (9)” or “100” as answer option or answering “no” to the honesty question. For the purpose of this study, individuals reporting having a psychiatric diagnosis or taking psychotropic medication (*n* = 76) were also removed. The final sample includes data of 440 participants. See Table 1 in the main text for an overview of the sample size per measure.

# Measures

**Smoking behavior and alcohol intake.**

Participants were asked to indicate whether they are a “smoker”, “smoke sometimes”, “non-smoker / only on a single occasion”, or “ex-smoker”. Alcohol consumption was assessed as frequency (“How often do you drink alcohol?”) and number of consumptions a week (“How many units of alcohol do you consume per week? (1 unit is 1/2 pint of average strength beer, a standard glass of wine is 2 units of alcohol)”.

**Sleep feature measures**

*Sleep duration deviation.* The participant’s deviation from the sample’s mean sleep duration was calculated for the last night’s sleep. Last night’s sleep duration was calculated from the variables time turned off light (“Yesterday I went to sleep (turned off the light) at ..”), minutes to fall asleep (“After I turned off the light, I fell asleep within .. (minutes)”), and wake time (“This morning I woke up at ..”). Higher values represent either shorter or longer sleep duration than the mean. Inverted U-shaped relationships are often found for sleep duration and health aspects, where both short and long sleep duration are associated with worse health ^3,4^.

*Social jetlag*. The Munich Chronotype Questionnaire (MCTQ) was used to estimate social jetlag, calculated as the difference in the midpoint of sleep on workdays and free days ^5^.

*Non-restorative sleep, poor sleep quality, perception of insufficient sleep.* These constructs were calculated using the Karolinska Sleep Questionnaire (KSQ) items ^6^. Items were rated on frequency in the past three months (from “0 never or seldom” to “4 times a week or more”). Non-restorative sleep was calculated as the mean score of “difficulty waking up” and “hard to stay awake during the day”. Poor sleep quality was calculated as the mean scores of “difficulty falling asleep” and “repeated awakenings with difficulty falling asleep again”. The perception of insufficient sleep was measured by the response to the item “too little sleep”.

*Evening chronotype*. Circadian preference, or chronotype, was assessed using the reduced Morningness-Eveningness Questionnaire (rMEQ) ^7^. The continuous rMEQ score is used, rescored such that a higher score is interpretated as a tendency towards being an evening-type. A subset of the participants (n = 114, 25.9%) inaccurately completed the rMEQ item “At what time in the evening do you feel tired and as a result in need of sleep?”, likely due to misunderstanding the question and/or answer option. This item was therefore omitted from the rMEQ calculation for these individuals. Their rMEQ score was recalculated based on the remaining four items (range 3-21) and aligned with the 4-25 range, consistent with the possible range of the 5-item rMEQ).

*Fatigue.* Fatigue level was assessed using the item “I feel tired” from the Sickness Questionnaire ^8^.

*Insomnia*. The insomnia severity index (ISI) is a 7-item validated questionnaire for measuring insomnia symptoms, each item being rated from 0 = no problem to 4 = very severe problem, yielding a total score ranging from 0-28 ^9^.

See Figure S1 for distribution plots of the sleep variables and Figure S2 for a heatmap.

**Psychiatric trait questionnaires**

For all psychiatric trait questionnaires, a higher score is indicative of a higher psychiatric trait level. To align all questionnaires in terms of symptom time window, questionnaires that asked for symptoms in the past week or past two weeks were rephrased to ask for symptoms in the past month (CESD-R 10, GAD-7, ASRMS, ALS, OCI-R). See Figure S3 for distribution plots of the psychiatric trait scores and Figure S4 for a heatmap.

*Psychiatric trait questionnaires*

Thirteen validated questionnaires on common psychiatric traits and risk factors were included (referred to as “psychiatric traits” for the remainder of the text) assessing: depression; generalized anxiety; mania; delusional ideation; emotion dysregulation; autism; impulsivity; emotional instability; ADHD; OCD; eating disorder; apathy; social anxiety.

Depression. The Center for Epidemiologic Studies Depression Scale Revised Short Form (CESD-R 10) ^10^ is a 10-item questionnaire designed to measure depression in the general population. Two items are reverse scored. A total score is calculated by summing the ratings: 0 “rarely or none of the time”; 1 “some or a little of the time”; 2 “occasionally or a moderate amount of time”; 3 “almost all of the time”.

Generalized anxiety. The Generalized Anxiety Disorder-7 (GAD-7) ^11^ is a 7-item questionnaire designed to identify probable cases of GAD. The ratings (0, 1, 2, or 3) are added to retrieve a total score. Total scores of 5, 10, and 15 are taken as the cut-off points for mild, moderate, and severe anxiety, respectively.

Mania. The 5-item Altman Self-Rating Mania Scale (ASRMS) ^12^ is designed to assess the presence and severity of manic symptoms. A total score is calculated by summing the item ratings (0, 1, 2, 3, 4). A cutoff score of 6 or higher serves as an indication for further (hypo)mania assessment.

Delusional ideation. Delusions (yes/no subscale of Peters Delusions Inventory 21 (PDI-21) ^13^ and unusual experiences subscale of the Oxford-Liverpool Inventory of Feelings and Experiences (O-LIFE) ^14^ were used to assess delusional ideation. The PDI-21 was designed to measure schizotypal traits in the general population. The combined PDI-21 and O-LIFE unusual experiences subscale consisted of 33 yes/no items, asking for occurrence of experiences during their lifetime, such as “do you every feel that there is a conspiracy against you?” and “are your thoughts sometimes so strong that you can almost hear them?”. Delusional ideation was scored by summing the count of all endorsements (“yes” responses).

Emotion dysregulation. The difficulties in emotion regulation scale (DERS-16) ^15^ is a 16-item questionnaire designed to measure overall emotion regulation difficulties. The total score is obtained by summing the ratings (1, 2, 3, 4, 5) of each item.

Autism. The Autism Quotient-10 (AQ-10) ^16^ is a questionnaire to measure the extent of autistic traits in adults. Items are rated on a 4-point scale. The items are scored with 0 or 1. The answer options “definitely agree” and “slightly agree” are scored as 1 for item 1, 7, 8, and 10, while “definitely disagree” and “slightly disagree” are scored as 1 for the other items. Item ratings are summed to receive a total score. A cut-off score of 6 or higher indicates a significant number of autistic traits.

Impulsivity. The 20-item Health-relevant Personality Inventory (HP5i) ^17^ measures five health-relevant personality traits. The impulsivity subscale was used for this study. Items are rated on a 4-point scale with answer options “does not apply at all”, “does not apply very well”, “applies pretty much” and “applies completely”. The impulsivity score is calculated by averaging the four items.

Emotional instability. The Affective Lability Scale (ALS-18) ^18^ is an 18-item questionnaire designed to measure rapid shifts in outward emotional expressions, i.e., emotional instability. The items are rated on a 4-point scale from 0 to 3 (“very uncharacteristic of me” to “very characteristic of me”) and a total score is calculated by summing the ratings.

ADHD. The Adult ADHD Self-Report Scale (ASRS) ^19^ is a screening questionnaire for use in the general population. The questionnaire consists of 18 items assessing the frequency of DSM-IV Criterion A symptoms of adult ADHD. Each item is rated on a 5-point scale. The items were rescored dichotomously (0 or 1), with a score of 1 assigned to items rated as “often” or “very often” for 11 items, and for the remaining seven items a score of 1 was assigned for “sometimes”, “often”, or “very often”. The total score is calculated by summing the scores.

OCD. The Obsessive-Compulsive Inventory-Revised (OCI-R) ^20^ is an 18-item questionnaire to measure the severity and type of OCD symptoms present, rated on a 5-point scale from 0 “not at all” to 4 “extremely”. The total score is calculated by summing the item ratings.

Eating disorder. Part B of the Eating Attitudes Test (EAT-26) ^21^ was completed to measure eating disorder trait. This 26-item questionnaire contains items on eating-related attitudes, feelings, and behaviors, that are rated on a 6-point scale from “always” scored as 3, “usually” as 2, “often” as 1, and “sometimes”, “rarely” and “never” as 0. One item is reverse scored. The total score is calculated by summing the ratings.

Apathy. The Apathy Evaluation Scale (AES) ^22^ measures behavioral, cognitive, and emotional concomitants of deficits in goal-directed behavior that reflect apathy. This 18-item questionnaire is scored on a 4-point scale, from 1 “not at all” to 4 “a lot”. Three items are reverse scored and the total score is calculated by summing the rating of all items.

Social anxiety. The Liebowitz Social Anxiety Scale (LSAS) ^23,24^ assesses fear/anxiety and avoidance of common situations. The 24 items are rated twice. Once on fear or anxiety, rated from 0 “none” (fear or anxiety) to 3 “severe” (fear or anxiety) and once on avoidance, rated from 0 “never (0%)” to 3 “usually (67-100%)”. The total score is calculated by summing the ratings of both subscales.

Other measures that are not included in the analysis of the current study are measures of emotional and perceptual judgements, a social metacognition task, and questionnaires regarding sickness symptoms and beliefs in COVID conspiracy theories.

# ADDITIONAL RESULTS

# Table S1. Sample characteristics: smoking behavior and alcohol intake.

|  | **N (%)** |
| --- | --- |
| Smoking behavior |  |
| Smoker | 27 (6.1%) |
| Smoke sometimes | 29 (6.6%) |
| Non-smoker / only on a single occasion | 279 (63.4%) |
| Ex-smoker | 105 (23.9%) |
| Alcohol – frequency |  |
| 4 times a week or more | 14 (3.2%) |
| 2-3 times a week | 59 (13.4%) |
| 2-4 times a month | 124 (28.2%) |
| On occasion, once a month or more rarely | 165 (37.5%) |
| Never | 78 (17.7%) |
| Alcohol – consumptions a week* |  |
| >20 units | 5 (1.1%) |
| 16-20 units | 8 (1.8%) |
| 11-15 units | 24 (5.5%) |
| 6-10 units | 49 (11.1%) |
| 1-5 units | 174 (39.6%) |
| 0 | 180 (40.9%) |

*Note. *1 unit is 1/2 pint of average strength beer; a standard glass of wine is 2 units of alcohol.*

**SUPPLEMENTARY TABLES**

For Table S2-14, positive regression coefficients indicate that as psychiatric trait levels are higher, there is a corresponding increase in insomnia severity, heightened fatigue levels, greater non-restorative sleep, worse sleep quality, stronger perception of insufficient sleep, stronger eveningness, greater deviation in sleep duration (either shorter or longer sleep duration than the mean of 7h39min), and greater social jetlag.

# Table S2. Regression results for the sleep features and ASRS (Adult ADHD Self-Report Scale).

|  | | **ADHD (ASRS)** | | | |
| --- | --- | --- | --- | --- | --- |
|  | β | | 95% confidence interval | | *p* |
|  |  |  | Lower band | Upper band |  |
| Insomnia | **0.357** | | 0.262 | 0.451 | 0.000 |
| Fatigue | **0.365** | | 0.278 | 0.453 | 0.000 |
| Non-restorative sleep | **0.342** | | 0.248 | 0.437 | 0.000 |
| Poor sleep quality | **0.231** | | 0.133 | 0.329 | 0.000 |
| Perceived too little sleep | **0.258** | | 0.161 | 0.355 | 0.000 |
| Evening chronotype | **0.210** | | 0.118 | 0.302 | 0.000 |
| Sleep duration deviation | **0.097** | | 0.002 | 0.191 | 0.045 |
| Social jetlag | 0.022 | | -0.072 | 0.117 | 0.645 |

*Note.* Bold coefficients denote statistically significant effects (*p* < 0.05).

# Table S3. Regression results for the sleep features and AES (Apathy Evaluation Scale).

|  | | **Apathy (AES)** | | | |
| --- | --- | --- | --- | --- | --- |
|  | β | | 95% confidence interval | | *p* |
|  |  |  | Lower band | Upper band |  |
| Insomnia | **0.315** | | 0.220 | 0.409 | 0.000 |
| Fatigue | **0.351** | | 0.263 | 0.438 | 0.000 |
| Non-restorative sleep | **0.221** | | 0.125 | 0.318 | 0.000 |
| Poor sleep quality | **0.205** | | 0.108 | 0.302 | 0.000 |
| Perceived insufficient sleep | **0.242** | | 0.146 | 0.338 | 0.000 |
| Evening chronotype | **0.241** | | 0.150 | 0.332 | 0.000 |
| Sleep duration deviation | 0.051 | | -0.043 | 0.145 | 0.287 |
| Social jetlag | -0.066 | | -0.160 | 0.029 | 0.173 |

*Note.* Bold coefficients denote statistically significant effects (*p* < 0.05).

# Table S4. Regression results for the sleep features and AQ10 (Autism Quotient-10).

|  | | **Autism (AQ-10)** | | | |
| --- | --- | --- | --- | --- | --- |
|  | β | | 95% confidence interval | | *p* |
|  |  |  | Lower band | Upper band |  |
| Insomnia | **0.134** | | 0.028 | 0.239 | 0.013 |
| Fatigue | **0.158** | | 0.066 | 0.251 | 0.001 |
| Non-restorative sleep | **0.124** | | 0.019 | 0.230 | 0.021 |
| Poor sleep quality | 0.092 | | -0.014 | 0.197 | 0.090 |
| Perceived insufficient sleep | 0.048 | | -0.058 | 0.154 | 0.376 |
| Evening chronotype | **0.182** | | 0.090 | 0.274 | 0.000 |
| Sleep duration deviation | 0.044 | | -0.050 | 0.138 | 0.361 |
| Social jetlag | 0.083 | | -0.011 | 0.177 | 0.084 |

*Note.* Bold coefficients denote statistically significant effects (*p* < 0.05).

# Table S5. Regression results for the sleep features and PDI-21 & O-LIFE subscale.

|  | | **Delusional ideation (PDI-21 & O-LIFE subscale)** | | | |
| --- | --- | --- | --- | --- | --- |
|  | β | | 95% confidence interval | | *p* |
|  |  |  | Lower band | Upper band |  |
| Insomnia | **0.170** | | 0.068 | 0.271 | 0.001 |
| Fatigue | **0.213** | | 0.121 | 0.305 | 0.000 |
| Non-restorative sleep | **0.263** | | 0.164 | 0.363 | 0.000 |
| Poor sleep quality | 0.096 | | -0.006 | 0.199 | 0.066 |
| Perceived insufficient sleep | 0.044 | | -0.059 | 0.147 | 0.399 |
| Evening chronotype | **0.154** | | 0.062 | 0.247 | 0.001 |
| Sleep duration deviation | **0.115** | | 0.021 | 0.209 | 0.017 |
| Social jetlag | **0.107** | | 0.013 | 0.201 | 0.025 |

# *Note.* PDI-21 = Peters Delusions Inventory 21; O-LIFE = Oxford-Liverpool Inventory of Feelings and Experiences (unusual experiences subscale). Bold coefficients denote statistically significant effects (*p* < 0.05).

# Table S6. Regression results for the sleep features and CESD-R-10 (Center for Epidemiologic Studies Depression Scale – Revised 10 item).

|  | | **Depression (CESD-R 10)** | | | |
| --- | --- | --- | --- | --- | --- |
|  | β | | 95% confidence interval | | *p* |
|  |  |  | Lower band | Upper band |  |
| Insomnia | **0.575** | | 0.491 | 0.659 | 0.000 |
| Fatigue | **0.507** | | 0.426 | 0.588 | 0.000 |
| Non-restorative sleep | **0.410** | | 0.317 | 0.503 | 0.000 |
| Poor sleep quality | **0.390** | | 0.296 | 0.484 | 0.000 |
| Perceived insufficient sleep | **0.412** | | 0.319 | 0.505 | 0.000 |
| Evening chronotype | **0.276** | | 0.186 | 0.367 | 0.000 |
| Sleep duration deviation | **0.153** | | 0.060 | 0.246 | 0.001 |
| Social jetlag | -0.038 | | -0.132 | 0.057 | 0.433 |

*Note.* Bold coefficients denote statistically significant effects (*p* < 0.05).

#

# Table S7. Regression results for the sleep features and EAT-26 part B (Eating Attitudes Test-26).

|  | | **Eating disorder (EAT-26 part B)** | | | |
| --- | --- | --- | --- | --- | --- |
|  | β | | 95% confidence interval | | *p* |
|  |  |  | Lower band | Upper band |  |
| Insomnia | **0.133** | | 0.031 | 0.235 | 0.011 |
| Fatigue | **0.242** | | 0.151 | 0.333 | 0.000 |
| Non-restorative sleep | **0.171** | | 0.069 | 0.272 | 0.001 |
| Poor sleep quality | **0.121** | | 0.019 | 0.223 | 0.020 |
| Perceived insufficient sleep | **0.105** | | 0.003 | 0.207 | 0.045 |
| Evening chronotype | 0.009 | | -0.085 | 0.103 | 0.847 |
| Sleep duration deviation | 0.030 | | -0.064 | 0.125 | 0.525 |
| Social jetlag | 0.002 | | -0.092 | 0.096 | 0.963 |

*Note.* Bold coefficients denote statistically significant effects (*p* < 0.05).

# Table S8. Regression results for the sleep features and DERS-16 (Difficulties in Emotion Regulation Scale-16).

|  | | **Emotion dysregulation (DERS-16)** | | | |
| --- | --- | --- | --- | --- | --- |
|  | β | | 95% confidence interval | | *p* |
|  |  |  | Lower band | Upper band |  |
| Insomnia | **0.371** | | 0.277 | 0.465 | 0.000 |
| Fatigue | **0.390** | | 0.304 | 0.476 | 0.000 |
| Non-restorative sleep | **0.319** | | 0.224 | 0.415 | 0.000 |
| Poor sleep quality | **0.308** | | 0.213 | 0.404 | 0.000 |
| Perceived insufficient sleep | **0.275** | | 0.179 | 0.372 | 0.000 |
| Evening chronotype | **0.202** | | 0.110 | 0.294 | 0.000 |
| Sleep duration deviation | 0.086 | | -0.008 | 0.180 | 0.073 |
| Social jetlag | 0.007 | | -0.087 | 0.101 | 0.880 |

*Note.* Bold coefficients denote statistically significant effects (*p* < 0.05).

# Table S9. Regression results for the sleep features and ALS-18 (Affective Lability Scale-18).

|  | | **Emotional instability (ALS-18)** | | | |
| --- | --- | --- | --- | --- | --- |
|  | β | | 95% confidence interval | | *p* |
|  |  |  | Lower band | Upper band |  |
| Insomnia | **0.389** | | 0.294 | 0.483 | 0.000 |
| Fatigue | **0.358** | | 0.270 | 0.446 | 0.000 |
| Non-restorative sleep | **0.365** | | 0.269 | 0.460 | 0.000 |
| Poor sleep quality | **0.288** | | 0.190 | 0.386 | 0.000 |
| Perceived insufficient sleep | **0.290** | | 0.192 | 0.388 | 0.000 |
| Evening chronotype | **0.234** | | 0.143 | 0.325 | 0.000 |
| Sleep duration deviation | **0.110** | | 0.016 | 0.204 | 0.022 |
| Social jetlag | **0.095** | | 0.001 | 0.189 | 0.048 |

*Note.* Bold coefficients denote statistically significant effects (*p* < 0.05).

# Table S10. Regression results for the sleep features and GAD-7 (Generalized Anxiety Disorder-7).

|  | | **Generalized anxiety (GAD-7)** | | | |
| --- | --- | --- | --- | --- | --- |
|  | β | | 95% confidence interval | | *p* |
|  |  |  | Lower band | Upper band |  |
| Insomnia | **0.442** | | 0.351 | 0.534 | 0.000 |
| Fatigue | **0.429** | | 0.344 | 0.514 | 0.000 |
| Non-restorative sleep | **0.315** | | 0.219 | 0.412 | 0.000 |
| Poor sleep quality | **0.376** | | 0.281 | 0.470 | 0.000 |
| Perceived insufficient sleep | **0.368** | | 0.273 | 0.462 | 0.000 |
| Evening chronotype | **0.168** | | 0.075 | 0.260 | 0.000 |
| Sleep duration deviation | **0.103** | | 0.008 | 0.197 | 0.033 |
| Social jetlag | -0.028 | | -0.122 | 0.066 | 0.562 |

*Note.* Bold coefficients denote statistically significant effects (*p* < 0.05).

# Table S11. Regression results for the sleep features and HP5i impulsivity subscale (Health-relevant Personality Inventory).

|  | | **Impulsivity (HP5i subscale)** | | | |
| --- | --- | --- | --- | --- | --- |
|  | β | | 95% confidence interval | | *p* |
|  |  |  | Lower band | Upper band |  |
| Insomnia | **0.179** | | 0.080 | 0.279 | 0.000 |
| Fatigue | **0.128** | | 0.035 | 0.221 | 0.007 |
| Non-restorative sleep | **0.172** | | 0.072 | 0.271 | 0.001 |
| Poor sleep quality | **0.139** | | 0.039 | 0.238 | 0.007 |
| Perceived insufficient sleep | 0.080 | | -0.021 | 0.180 | 0.119 |
| Evening chronotype | **0.116** | | 0.023 | 0.209 | 0.015 |
| Sleep duration deviation | 0.039 | | -0.055 | 0.134 | 0.412 |
| Social jetlag | 0.036 | | -0.058 | 0.130 | 0.452 |

*Note.* Bold coefficients denote statistically significant effects (*p* < 0.05).

# Table S12. Regression results for the sleep features and ASRMS (Altman Self-Rating Mania Scale).

|  | | **Mania (ASRMS)** | | | |
| --- | --- | --- | --- | --- | --- |
|  | β | | 95% confidence interval | | *p* |
|  |  |  | Lower band | Upper band |  |
| Insomnia | **-0.204** | | -0.301 | -0.107 | 0.000 |
| Fatigue | **-0.170** | | -0.263 | -0.078 | 0.000 |
| Non-restorative sleep | **-0.108** | | -0.206 | -0.010 | 0.031 |
| Poor sleep quality | -0.095 | | -0.194 | 0.003 | 0.057 |
| Perceived insufficient sleep | **-0.188** | | -0.285 | -0.091 | 0.000 |
| Evening chronotype | **-0.137** | | -0.230 | -0.044 | 0.004 |
| Sleep duration deviation | -0.033 | | -0.128 | 0.062 | 0.492 |
| Social jetlag | 0.031 | | -0.063 | 0.125 | 0.522 |

*Note.* Bold coefficients denote statistically significant effects (*p* < 0.05).

# Table S13. Regression results for the sleep features and OCI-R (Obsessive Compulsive Inventory-Revised).

|  | | **OCD (OCI-R)** | | | |
| --- | --- | --- | --- | --- | --- |
|  | β | | 95% confidence interval | | *p* |
|  |  |  | Lower band | Upper band |  |
| Insomnia | **0.277** | | 0.180 | 0.375 | 0.000 |
| Fatigue | **0.267** | | 0.177 | 0.358 | 0.000 |
| Non-restorative sleep | **0.312** | | 0.216 | 0.408 | 0.000 |
| Poor sleep quality | **0.165** | | 0.065 | 0.265 | 0.001 |
| Perceived insufficient sleep | **0.152** | | 0.052 | 0.253 | 0.003 |
| Evening chronotype | **0.168** | | 0.076 | 0.261 | 0.000 |
| Sleep duration deviation | **0.190** | | 0.097 | 0.282 | 0.000 |
| Social jetlag | **0.116** | | 0.023 | 0.209 | 0.015 |

*Note.* Bold coefficients denote statistically significant effects (*p* < 0.05).

# Table S14. Regression results for the sleep features and LSAS (Liebowitz Social Anxiety Scale).

|  | | **Social anxiety (LSAS)** | | | |
| --- | --- | --- | --- | --- | --- |
|  | β | | 95% confidence interval | | *p* |
|  |  |  | Lower band | Upper band |  |
| Insomnia | **0.284** | | 0.187 | 0.380 | 0.000 |
| Fatigue | **0.313** | | 0.224 | 0.402 | 0.000 |
| Non-restorative sleep | **0.306** | | 0.211 | 0.402 | 0.000 |
| Poor sleep quality | **0.298** | | 0.202 | 0.393 | 0.000 |
| Perceived insufficient sleep | **0.254** | | 0.157 | 0.351 | 0.000 |
| Evening chronotype | **0.153** | | 0.060 | 0.246 | 0.001 |
| Sleep duration deviation | 0.030 | | -0.064 | 0.124 | 0.528 |
| Social jetlag | 0.048 | | -0.046 | 0.143 | 0.316 |

*Note.* Bold coefficients denote statistically significant effects (*p* < 0.05).

**SUPPLEMENTARY FIGURES**

# Figure S1. Frequency plots of the scores of the sleep features.

# Figure S2. Correlation matrix showing Pearson correlation coefficients between the sleep feature measures.

# Figure S3. Frequency plots of the scores of the psychiatric trait levels. The black line denotes the available cut-off scores for potential clinical-level symptoms: Depression ≥ 10; Generalized anxiety ≥ 10 (= moderate anxiety); Mania ≥ 6; Autism ≥ 6; OCD (Obsessive Compulsive Disorder) ≥ 21; Eating disorder ≥ 20; Apathy ≥ 38; Social anxiety ≥ 50 (= moderate social anxiety); No cut-off values are available or applicable for all other questionnaires.

# Figure S4. Correlation matrix showing Pearson correlation coefficients between the psychiatric trait questionnaires.

# REFERENCES

1 Peer E, Rothschild D, Gordon A, Evernden Z, Damer E. Data quality of platforms and panels for online behavioral research. *Behavior Research Methods* 2022; **54**: 1643–1662.

2 Oppenheimer DM, Meyvis T, Davidenko N. Instructional manipulation checks: Detecting satisficing to increase statistical power. *Journal of Experimental Social Psychology* 2009. doi:10.1016/j.jesp.2009.03.009.

3 Andreasson A, Axelsson J, Bosch JA, Balter LJ. Poor sleep quality is associated with worse self-rated health in long sleep duration but not short sleep duration. *Sleep Medicine* 2021; **88**: 262–266.

4 Wang YH, Wang J, Chen SH, Li JQ, Lu QD, Vitiello MV *et al.* Association of Longitudinal Patterns of Habitual Sleep Duration with Risk of Cardiovascular Events and All-Cause Mortality. *JAMA Network Open* 2020; **3**: e205246.

5 Roenneberg T, Wirz-Justice A, Merrow M. Life between clocks: Daily temporal patterns of human chronotypes. *Journal of Biological Rhythms* 2003; **18**: 80–90.

6 Nordin M, Åkerstedt T, Nordin S. Psychometric evaluation and normative data for the karolinska sleep questionnaire. *Sleep and Biological Rhythms* 2013; **11**: 216–226.

7 Adan A, Almirall H. Horne & Östberg morningness-eveningness questionnaire: A reduced scale. *Personality and Individual Differences* 1991; **12**: 241–253.

8 Andreasson A, Wicksell RK, Lodin K, Karshikoff B, Axelsson J, Lekander M. A global measure of sickness behaviour: Development of the Sickness Questionnaire. *Journal of Health Psychology* 2018; **23**: 1452–1463.

9 Bastien CH, Vallières A, Morin CM. Validation of the insomnia severity index as an outcome measure for insomnia research. *Sleep Medicine* 2001; **2**: 297–307.

10 Cole JC, Rabin AS, Smith TL, Kaufman AS. Development and validation of a Rasch-derived CES-D short form. *Psychological Assessment* 2004; **16**: 360–372.

11 Löwe B, Decker O, Müller S, Brähler E, Schellberg D, Herzog W *et al.* Validation and standardization of the generalized anxiety disorder screener (GAD-7) in the general population. *Medical Care* 2008; **46**: 266–74.

12 Altman EG, Hedeker D, Peterson JL, Davis JM. The altman self-rating Mania scale. *Biological Psychiatry* 1997; **42**: 948–955.

13 Peters E, Joseph S, Day S, Garety P. Measuring delusional ideation: The 21-item Peters et al. Delusions Inventory (PDI). *Schizophrenia Bulletin* 2004; **30**: 1005–22.

14 Mason O, Claridge G. The Oxford-Liverpool Inventory of Feelings and Experiences (O-LIFE): Further description and extended norms. *Schizophrenia Research* 2006; **82**: 203–211.

15 Bjureberg J, Ljótsson B, Tull MT, Hedman E, Sahlin H, Lundh LG *et al.* Development and Validation of a Brief Version of the Difficulties in Emotion Regulation Scale: The DERS-16. *Journal of Psychopathology and Behavioral Assessment* 2016; **38**: 284–296.

16 Booth T, Murray AL, McKenzie K, Kuenssberg R, O’Donnell M, Burnett H. Brief report: An evaluation of the AQ-10 as a brief screening instrument for asd in adults. *Journal of Autism and Developmental Disorders* 2013; **43**: 2997–3000.

17 Gustavsson JP, Jönsson EG, Linder J, Weinryb RM. The HP5 inventory: Definition and assessment of five health-relevant personality traits from a five-factor model perspective. *Personality and Individual Differences* 2003; **35**: 69–89.

18 Oliver MNI, Simons JS. The affective lability scales: Development of a short-form measure. *Personality and Individual Differences* 2004; **37**: 1279–1288.

19 Adler LA, Spencer T, Faraone SV, Kessler RC, Howes MJ, Biederman J *et al.* Validity of pilot adult ADHD Self-Report Scale (ASRS) to rate adult ADHD symptoms. *Annals of Clinical Psychiatry* 2006; **18**: 145–8.

20 Foa EB, Huppert JD, Leiberg S, Langner R, Kichic R, Hajcak G *et al.* The Obsessive-Complusive Inventory: Development and validation of a short version. *Psychological Assessment* 2002; **14**: 485–96.

21 Garner DM, Bohr Y, Garfinkel PE. The Eating Attitudes Test: Psychometric Features and Clinical Correlates. *Psychological Medicine* 1982; **12**: 871–878.

22 Marin RS, Biedrzycki RC, Firinciogullari S. Reliability and validity of the apathy evaluation scale. *Psychiatry Research* 1991; **38**: 143–62.

23 Heimberg RG, Horner KJ, Juster HR, Safren SA, Brown EJ, Schneier FR *et al.* Psychometric properties of the Liebowitz Social Anxiety Scale. *Psychological Medicine* 1999; **29**: 199–212.

24 Liebowitz MR. Liebowitz Social Anxiety Scale. *Modern Problems of Pharmapsychiatry* 1987.
